# Dissecting Genetic Diversity and Evolutionary Trends of Chinese PRRSV-1 Based on Whole-Genome Analysis

**DOI:** 10.1155/2024/9705539

**Published:** 2024-06-11

**Authors:** Bangjun Gong, Hu Xu, Qi Sun, Chao Li, Lirun Xiang, Jing Zhao, Wansheng Li, Zhenyang Guo, Jinhao Li, Qian Wang, Jinmei Peng, Guohui Zhou, Chaoliang Leng, Yan-Dong Tang, Jianan Wu, Huairan Liu, Tong-Qing An, Xuehui Cai, Zhi-Jun Tian, Hongliang Zhang

**Affiliations:** ^1^ State Key Laboratory for Animal Disease Control and Prevention Harbin Veterinary Research Institute Chinese Academy of Agricultural Sciences Harbin 150001China; ^2^ Henan Provincial Engineering and Technology Center of Animal Disease Diagnosis and Integrated Control Nanyang Normal University Nanyang 473061China

## Abstract

Porcine reproductive and respiratory syndrome (PRRS) poses a serious threat to the Chinese swine industry. The etiological agent PRRSV can be classified as either PRRSV-1 or PRRSV-2. Recent studies have revealed an increase in the rates of PRRSV-1 detection and a wider PRRSV-1 distribution. However, the PRRSV-1 genome in China has yet to be fully characterized. In this study, 24 whole PRRSV-1 genomes from different swine farms were assembled and subjected to whole-genome analysis. A phylogenetic analysis based on the complete genome and ORF5 sequences revealed that the PRRSV-1 strains from China belonged to Western European Subtype I and could be classified into seven subgroups. Statistical analysis revealed that BJEU06-1-Like PRRSV is currently the predominant PRRSV-1 strain. Moreover, a similarity analysis showed low pairwise similarity between most PRRSV-1 genomes from different pig farms. Amino acid alignments of the Nsp2 gene revealed that the BJEU06-1-Like subgroup had five discontinued aa deletions (4 + 1). The new subgroup 1 had 11 continued aa deletions and an aa insertion, the new subgroup 2 had two discontinued aa deletions (1 + 1), and, except for in the case of HKEU16, the HKEU16-Like subgroup had five discontinuous aa deletions (1 + 4). Recombination analysis revealed that the BJEU06-1-Like and NMEU09-1-Like strains participated extensively in recent recombination events. The analysis of positive selection suggested that there were 15 positively selected codons in site model, and there were five sites under positive selection in the BJEU06-1-Like subgroup in the branch-site model. The mean rate and tMRCA for PRRSV-1 strains from China were 4.11 × 10^−3^ substitutions/site/year and 1,969.63, respectively. Thus, it is crucial to strengthen epidemiological surveys of PRRSV-1 in China, especially those monitoring BJEU06-1-Like PRRSV.

## 1. Introduction

Porcine reproductive and respiratory syndrome (PRRS) has been highly detrimental to the swine industry since it became prevalent in North America in 1987 [1] and in western Europe in 1990 [2]. PRRS virus, the pathogen that causes PRRS, is an enveloped and positive single-strand RNA virus of the Arteriviridae family in the Nidovirales order [3]. The whole PRRSV genome is approximately 15 kb in length and comprises a 5′ cap structure, 5′ untranslated region (UTR), more than 10 open reading frames (ORFs), 3′ UTR, and a 3′ poly(A) tail. ORF1a and ORF1b, accounting for two-thirds of the virus genome, encode a large replicase polyprotein involved in viral replication and transcription that can hydrolyze more than 16 nonstructural proteins (Nsp1*α*, Nsp1*β*, Nsp2N, Nsp2TF, Nsp2-6, Nsp7*α*, Nsp7*β*, and Nsp8-12), while the remaining ORFs following ORF1b encode structural proteins relevant to virions (GP2a, E, GP3, GP4, GP5, ORF5a, M, and N) [4, 5, 6]. PRRSV has been taxonomically divided into *Betaarterivirus suid 1* and *Betaarterivirus suid 2*, with the representative isolates being Lelystad virus and ATCC VR-2332, respectively [7]. PRRSV-2 can be classified into nine lineages [8]. Previous studies have suggested that PRRSV-1 can be classified into four subtypes: Western European Subtype I, Russian Subtype I, Subtype II, and Subtype III [8]. All PRRSV-1 strains from China have been classified as Western European Subtype I and divided into four subgroups (Amervac-Like, BJEU06-1-Like, HKEU16-Like, and NMEU09-1-Like subgroups) on the basis of a complete-genome tree [9]. The pathogenicity of PRRSV-1 strains from China is generally low, but some strains exhibit moderate pathogenicity [10, 11, 12, 13, 14, 15]. The recombination patterns of PRRSV-1 strains from China are also quite complex. Moreover, recombinant strains originating from wild-type to wild-type, vaccine strain to vaccine strain, and wild-type to vaccine strain recombination events have all been detected [9, 16, 17].

Many studies have demonstrated the distribution and molecular characteristics of PRRSV-1 strains from countries other than China [18, 19, 20]. However, due to the limited availability of complete genome data for PRRSV-1 in public databases, few studies have systematically characterized the whole genome of PRRSV-1 strains from China [9, 21, 22]. Given the continuous increase in the detection of PRRSV-1 in China, we sequenced 24 whole genomes of Chinese PRRSV-1 strains and explored their characteristics by performing phylogenetic analysis, amino acid alignment, recombination analysis, positive selection analysis, and estimation of the evolutionary rate.

## 2. Materials and Methods

### 2.1. Clinical Sample Collection and Complete Genome Sequencing

Twenty-four PRRSV-1-positive specimens were collected from different pig farms and stored at the Harbin Veterinary Research Institute. Tissue specimen disposal, RNA extraction, reverse transcription polymerase chain reaction (RT-PCR), and genome sequencing were performed as previously described [23, 24]. The primers used to amplify the whole genome were also described previously [15, 25]. Lasergene software (DNASTAR Inc., Madison, USA) was used to assemble the PRRSV-1 sequences to generate 24 complete genomes, the sequence data for which have been deposited in the GenBank database with the accession numbers PP330948-PP330950, PP336343-PP336346, PP341288-PP341290, PP350850-PP350855, and PP402109-PP402114.

### 2.2. Phylogenetic Analysis

A total of 2,375 reference sequences were downloaded from the National Center for Biotechnology Information (NCBI) database. All sequences used to infer phylogenetic trees were aligned and trimmed using MAFFT v.7.471 and TrimAl v.1.2 rev57 wrapped in PhyloSuite with the default parameters [26, 27, 28]. Phylogenetic trees based on ORF5and the whole genome were inferred using IQ-TREE under the GTR + R5 + F, TVM + R10 + F, and GTR + R4 + F models for 20,000 ultrafast bootstraps, as well as the Shimodaira–Hasegawa-like approximate likelihood ratio test [29, 30, 31]. Phylogenetic trees based on the Nsp2 gene were constructed using the neighbor-joining method with 1,000 bootstrap replicates in MEGA 6.0 [32]. The putative amino acid sequences were aligned by ClustalW in MEGA 6.0 [32].

### 2.3. Recombination Analysis

Possible recombination events were detected by utilizing seven algorithms (3Seq, GeneConv, MaxChi, Chimera, RDP, SiScan, and BootScan) with the default parameters in RDP4 [33]. Only the recombination events identified by at least four of the seven algorithms were retained [34]. Then, the possible recombination events were verified by NCBI BLAST and SimPlot v.3.5.1 (boot scanning analysis was performed with a 200 bp window, sliding along the genome alignment with a step size of 20 bp) [34, 35]. Finally, the recombination events were validated using phylogenetic trees constructed by the neighbor-joining method in MEGA 6.0 [34].

### 2.4. Codon-Based Analyses of Positive Selection in GP5

In the site models, five different methods were applied to assess whether there were codons under selection. Only sites identified by at least three of the five methods were regarded as being under positive selection [36]. The alignment of the PRRSV-1 ORF5 gene was first subjected to analysis in PAML v4.4 to identify positively selected sites [37]. Likelihood ratio tests (LRTs) were performed to validate whether the alternative models (M2a or M8) fit better than the null models (M1a or M7) [36]. The M8 model was used to identify sites under positive selection. The Bayes empirical Bayes (BEB) method was utilized to verify codons with a posterior probability > 90% [38]. Then, the alignment was also analyzed with SLAC, FEL, MEME, and FUBAR [39, 40]. Only sites with *p* values < 0.1 for the SLAC, FEL, and MEME analyses and a posterior probability > 0.90 for the FUBAR analysis were considered candidates for positive selection [36]. Moreover, the branch-site model was also applied to identify positive selection that affects only some sites on prespecified lineages using PAML v4.4 [36]. The LRTs were also calculated to validate whether the alternative models (model A) fit better than the null models (model A with *ω*_2_ = 1) [36]. The posterior probability of these specific sites was also calculated via the BEB approach [38].

### 2.5. Evolutionary Dynamics Analysis

The rate of evolution and the time to the most recent common ancestor (tMRCA) for PRRSV-1 strains from China, and BJEU06-Like PRRSV were independently estimated from 60 Chinese PRRSV-1ORF5 sequence sets that were collected from different swine or had a lower pairwise nucleotide similarity (approximately 99.5%) using the coalescent-based Bayesian Markov chain Monte Carlo (MCMC) method [41]. Bayesian MCMC inference was applied under the best-fit molecular clock and coalescent tree prior models, which were determined by both path sampling and stepping-stone sampling procedures [41, 42, 43]. Three independent runs (200 million chain lengths and logging parameters every 20,000 iterations) were performed. Three log files and three tree files were combined with the proper burn-in function in LogCombiner (version 1.10.4). The combined log files were subsequently analyzed in Tracer (version 1.7.2) to evaluate the convergence (effective sample size > 200). The maximum clade credibility (MCC) tree was generated by TreeAnnotator v1.10.4 [43].

## 3. Results

### 3.1. Epidemic Status and Whole-Genome Sequencing for Chinese PRRSV-1

**Figure 1:**
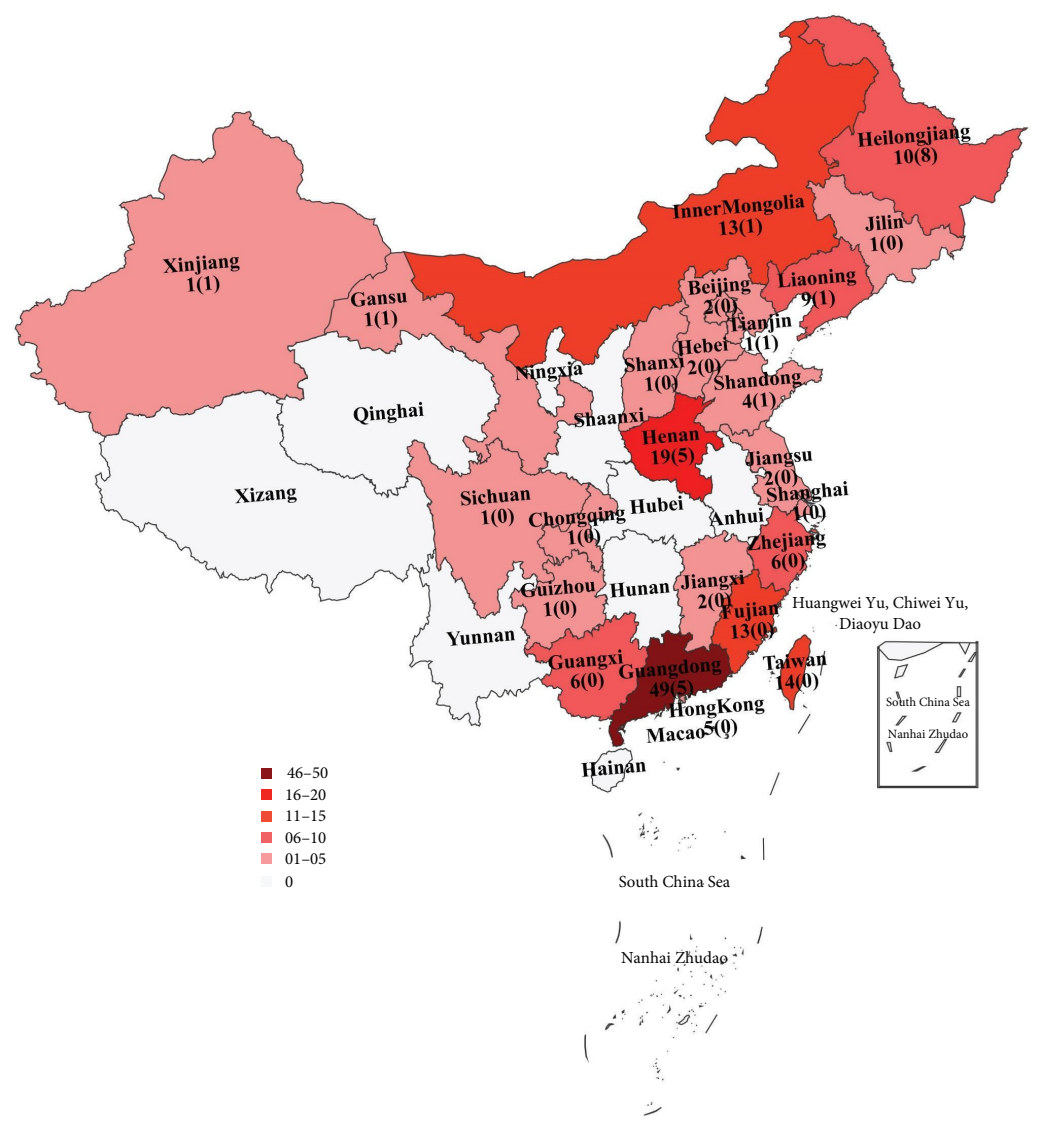
The geographical distribution of Chinese PRRSV-1. PRRSV-1 has been reported in a minimum of 24 provinces and regions at present. The number outside the parentheses represents the minimum number of reported cases of PRRSV-1 in the corresponding province/region at present, while the number inside represents the number of strains included in this research. This map was generated with pyecharts v.1.9.1.

Our epidemiological investigation revealed a significant increase in the quantity and geographical distribution of PRRSV-1 in recent years. Currently, PRRSV-1 has been detected in at least 24 provinces and regions in China (Figure 1). To elucidate the genome characteristics of PRRSV-1 strains identified in China in recent years, we conducted whole-genome sequencing on 24 positive samples collected from nine provinces and regions (Figure 1). These complete viral genomes ranged in length from 14,871 to 15,083 nt, excluding polyA, and exhibited 84.9%–88.9% nucleotide identity to the Lelystad virus but 59.9%–60.9% nucleotide identity to the ATCC VR2332 isolate.

### 3.2. Phylogenetic Analysis of Chinese PRRSV-1

**Figure 2:**
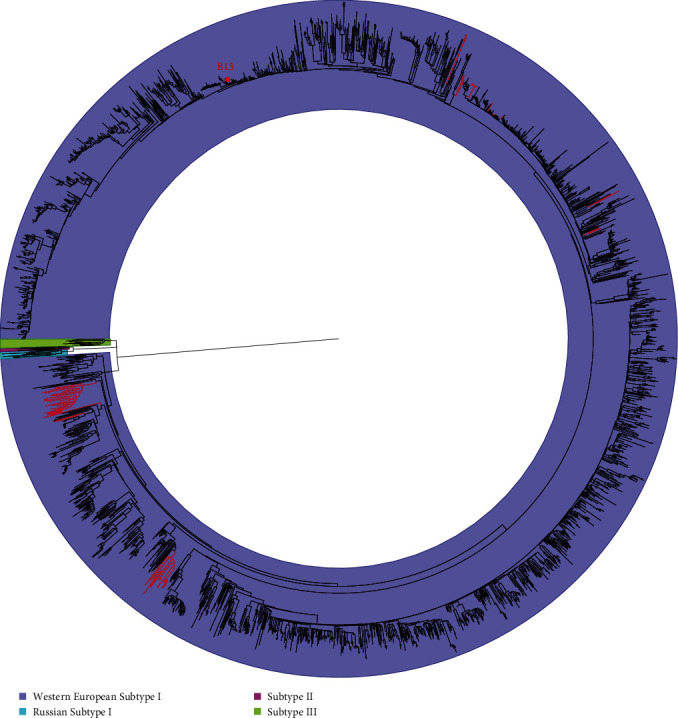
Maximum likelihood phylogeny of PRRSV-1 based on the ORF5 gene. Western European Subtype I, Russian Subtype I, Subtype II, and Subtype III are shaded with pale purple, turquoise, pink, and light green, respectively. The PRRSV-1 strains from China are marked with red lines, while those in other countries are marked with black lines. The PRRSV-1 strains from China are distributed in Western European Subtype I.

**Figure 3:**
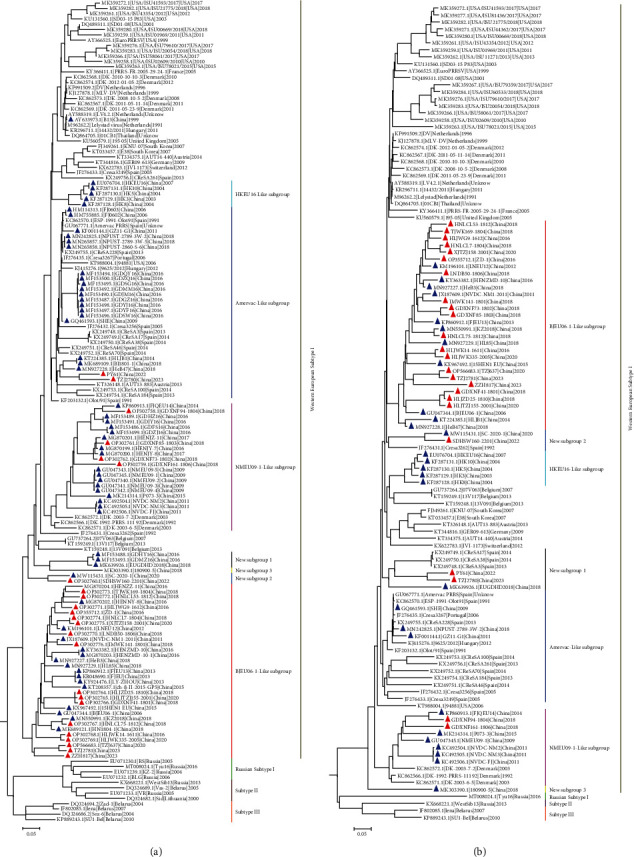
Phylogenic analysis of the ORF5 (a) and complete genome (b) nucleotide sequences of PRRSV-1 via the maximum likelihood method. The red solid triangles represent the 24 strains included in this study. Other PRRSV-1 strains from China isolates from the NCBI are marked by blue solid triangles. All the PRRSV-1 strains from China isolates were Western European Subtype I and could be divided into seven subgroups.

To investigate the genetic evolutionary relationship between PRRSV-1 strains from China and other countries, a phylogenetic analysis based on the ORF5 gene (*n* = 2,377) was performed. The results showed that PRRSV-1 could be classified into four subtypes (Western European Subtype I, Russian Subtype I, Subtype II, and Subtype III), and all the PRRSV-1 strains from China were of the Western European Subtype I (Figure 2). To elucidate the classification of Chinese PRRSV-1, phylogenetic trees based on ORF5 (*n* = 155) and the complete genome (*n* = 117) were constructed. The two phylogenetic analysis results showed that the PRRSV-1 strains from Chinese isolates could be divided into seven subgroups (Figures 3(a) and 3(b)). According to the phylogenetic analysis based on the ORF5 sequence and complete genome, the 24 strains could be divided into four different subgroups, with BJEU06-1-Like PRRSV consistently predominant among the 24 strains. However, there was some phylogenetic divergence when inferring dendrograms based on different genes (Figures 3(a) and 3(b)).This phenomenon may reveal the occurrence of recombination events and complications associated with the PRRSV-1 genome.

### 3.3. Amino Acid and Nucleotide Similarity of PRRSV-1 Strains from China

**Figure 4:**
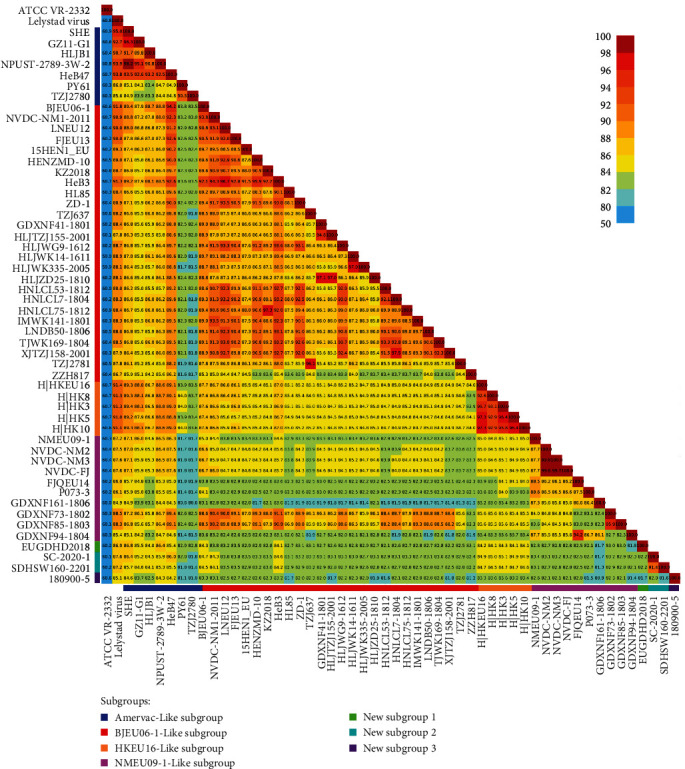
The pairwise similarity of the complete genomes of the PRRSV-1 strains from China and the representative strains of the two PRRSV genotypes. The color bar adjacent to the triangular matrix indicates the seven subgroups of PRRSV-1 in China, while the color bar on the right represents the gradient of similarity. This figure was drawn with matplotlib v.3.5.2.

To comprehensively understand the pairwise nucleotide similarity between different PRRSV-1 strains from China, we conducted a whole-genome nucleotide alignment. The results showed that there was a relatively low pairwise nucleotide similarity in the 24 complete genomes from different pig farms, with pairwise nucleotide similarities of 80%–90% accounting for 89.49%. A similar phenomenon was also observed for the pairwise nucleotide similarity of other complete Chinese PRRSV-1 strain genomes (Figure 4). These results indicated that there was significant genetic divergence in the complete PRRSV-1 genomes among different swine farms. To explore the nucleotide and amino acid similarity between the 24 strains and other reference strains, we first classified the 24 strains and other reference strains in a phylogenetic tree that was constructed based on ORF5 sequences (Figure 3(a)). We subsequently performed nucleotide and amino acid sequence alignments in a grouped manner. The nucleotide alignment results showed that ORF1a was more variable than the 3′ (5′) UTR and other ORFs between the 24 strains and reference strains of the seven subgroups (*Supplementary 1*). The nucleotide similarity between the NMEU09-1-Like subgroup strains in this study and the reference strains of the seven subgroups decreased to less than 80% at ORF1a (*Supplementary 1*). Amino acid alignment revealed that Nsp1*β* and Nsp2 were more variable than the other proteins between the 24 strains and the reference strains of the seven subgroups (*Supplementary 2*). The three subgroups in this study had low amino acid similarity (which decreased to below 80%) with the reference strains of the seven subgroups at Nsp1*β* and Nsp2 (*Supplementary 2*).

### 3.4. Indel Characteristics of Amino Acids in Chinese PRRSV-1 Strains

#### 3.4.1. Indel Characteristics of Amino Acids in Nsp2

**Figure 5:**
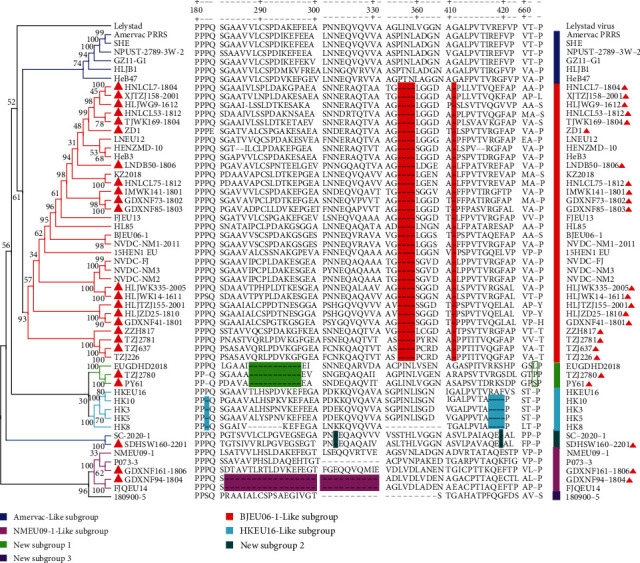
Amino acid alignment and phylogenetic analysis based on the Nsp2 gene. PRRSV-1 strains from China can be classified into seven subgroups based on the Nsp2 gene. The seven subgroups are marked by color bars and lines with deep blue, red, bright green, turquoise, cyan, pink, and purple colors. The red solid triangle represents the 24 strains identified in this study.

To explore the indel characteristics of Nsp2 amino acids, amino acid sequence alignment was performed. The results revealed that the BJEU06-1-Like subgroup had five discontinuous aa deletions (4 + 1) at positions 357–360 and 411. New subgroup 1 carried an 11-residue continued aa deletion at positions 288–298 and an aa insertion between the 661 and 662 sites. New subgroup 2 possessed two discontinuous aa deletions at positions 324 and 420 (1 + 1). Except for in the case of HKEU16, the HKEJU16-Like subgroup had five discontinuous aa deletions (1 + 4) at positions 182 and 418–421(Figure 5).

#### 3.4.2. Indel Characteristics of Amino Acids in GP3 and GP4

To understand the deletion features of GP3 and GP4, we conducted amino acid sequence analysis of GP3 and GP4. The results revealed that the C-terminus of GP3 has four deletion patterns and five premature termination patterns (*Supplementary 3*). The N-terminus of GP4 has four deletion patterns (*Supplementary 3*). However, there was no regular mutation in the overlapping region of GP3 and GP4 (*Supplementary 3*).

#### 3.4.3. Other Mutations in the Nsp12 and N Protein

Nsp12 originates from the C-terminus of the protein encoded by ORF1b and is relatively conserved compared to other Nsps. However, three strains exhibited premature termination (180900-5) or delayed termination (EUGDHD2018, HNLCL7-1804) at the C-terminus of Nsp12. The N protein of Western European Subtype I PRRSV commonly consists of 128 amino acids. In contrast, GDXNF85-1803 possessed a 4-aa truncation at the C-terminus of this protein, whereas NVDC-NM2 exhibited an aa insertion between the 87 and 88 sites.

### 3.5. Recombination Pattern of Chinese PRRSV-1

To identify the recombination events in the 24 strains, we used RDP4, Simplot, and BLAST to evaluate potential recombinant strains. Recombination events were detected in four of 24 complete genome sequences (Table 1). GDXNF41-1801 and HLJZD25-1810 resulted from recombination events between NMEU09-1 and BJEU06-1, with breakpoints ranging from the 5′UTR to Nsp1*β* (Table 1). GDXNF73-1802 and GDXNF85-1803 were derived from recombination events between NMEU09-1 and LNEU12 with breakpoints ranging from Nsp12 to ORF6 (Table 1). The four recombination events were also supported by phylogenetic trees (*Supplementary 4*). Currently, approximately 11 recombination events have been identified in Chinese PRRSV-1. Remarkably, recombination breakpoints mainly occurred in Nsps, and BJEU06-1-Like and NMEU09-1-Like strains extensively participated in recombination events (Table 1).

### 3.6. Selective Pressure on GP5 of Chinese PRRSV-1

**Figure 6:**
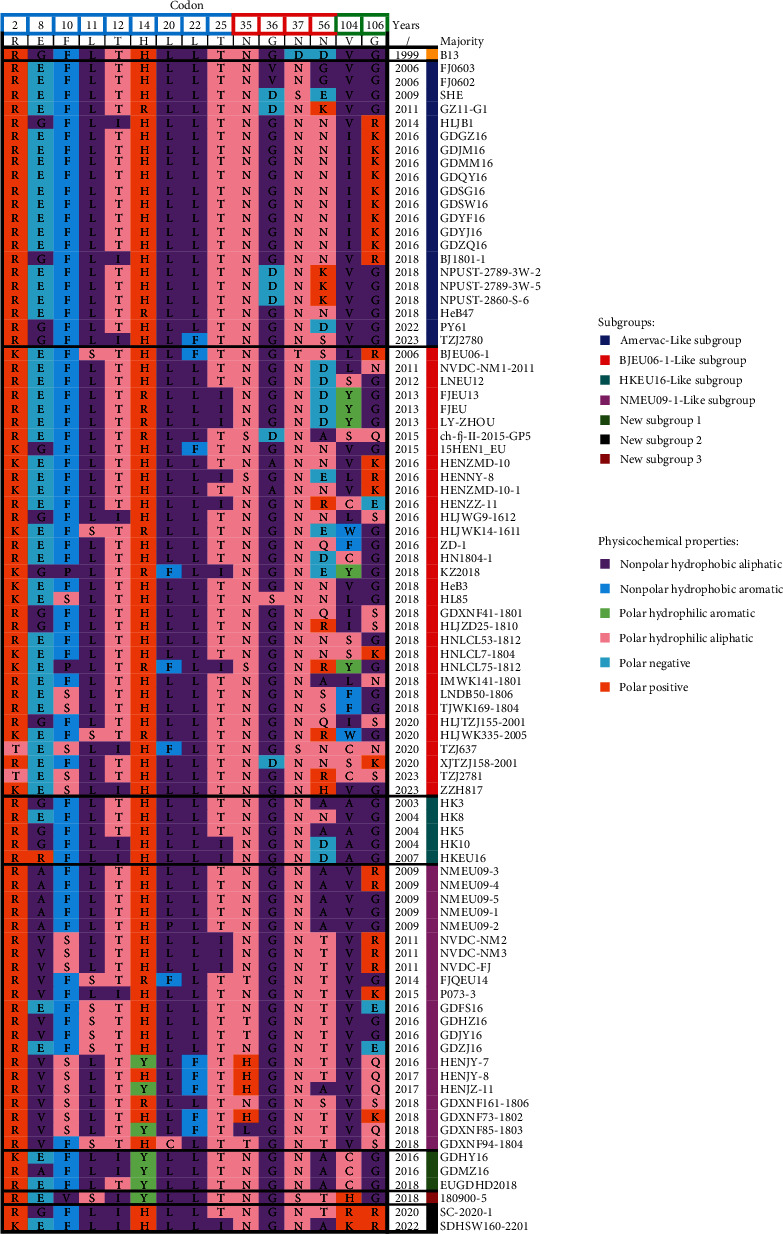
Positively selected codons and corresponding amino acid physicochemical properties for each PRRSV-1 strains from China based on the ORF5 gene. The 15 sites under positive selection were identified by at least three of the five different methods. Codons are numbered according to the deduced amino acid alignment of GP5. The filled colors represent the physicochemical properties of amino acids: nonpolar hydrophobic aliphatic (dark orchid), nonpolar hydrophobic aromatic (deep sky blue), polar hydrophilic aromatic (light green), polar hydrophilic aliphatic (light pink), polar negative (turquoise), and polar positive (chocolate). Positively selected sites in the signal peptide (SP), putative ectodomain 1 (EcD1), putative ectodomain 2 (EcD2), and endodomain (EnD) of PRRSV-1 are marked with light blue, red, and green squares, respectively.

To identify the positively selected codons in GP5 of PRRSV-1 strains from China in the site model, five different methods (PAML M8, SLAC, FEL, MEME, and FUBAR) were implemented separately. The comparison of M7 vs. M8 in PAML indicated significant LRTs (116.75 > *χ*_2,0.1%_^2^ = 10.83), with a *p* value < 0.001. Fifteen codons for GP5 were identified as potential sites under positive selection. These codons were distributed in the signal peptide (nine codons) and ectodomains (six codons) (Figure 6). In GP5, all positively selected sites exhibited at least one physicochemical alteration across strains (Figure 6). There were three codons (56, 104, and 106) with more combinations of amino acid physicochemical properties (Figure 6). Compared to other PRRSV-1 strains from China, the BJEU06-1-Like strains exhibited more combinations of amino acid physicochemical properties in positively selected codons (Figure 6). Thus, to determine whether positive selection affects some of the sites in the seven subgroups, the branch-site model was tested. The results showed that there were two subgroups with omega greater than 1, yet only the BJEU06-1-Like subgroup had statistically significant LRTs (87.61 > *χ*_1,0.1%_^2^ = 10.83), with a *p* value < 0.001 (Table 2). There were five positively selected codons (56, 60, 61, 104, and 106) in the BJEU06-1-Like subgroup, which had a probability higher than 95% (Table 2).

### 3.7. Evolutionary Rates and tMRCA of Chinese PRRSV-1

To estimate the rate of evolution and tMRCA of Chinese PRRSV-1, a time-scaled phylogenetic tree for the ORF5 gene was constructed under the best-fit molecular clock of uncorrelated, log normal distribution, and Bayesian skyline demographic model (*Supplementary 5*). Under that assumption, the rate of evolution of Chinese PRRSV-1 ORF5 was approximately 4.11 × 10^−3^ substitutions/site/year (95% HPD intervals: 2.29 × 10^−3^−6.01 × 10^−3^). The tMRCA of the Chinese PRRSV-1 strains was estimated to be approximately 1,969.63 (95% HPD range was 1,943.47–1,988.53) (*Supplementary 6*). To elucidate the nucleotide substitution rates of the seven subgroups, we counted the quantity of PRRSV-1 strains for every subgroup in the 60 Chinese PRRSV-1 ORF5 sequence sets. The results revealed that only the ORF5 sequences of BJEU06-1-Like PRRSV were suitable for Bayesian MCMC inference. Under the best-fit strict molecular clock and Bayesian skyline demographic model (*Supplementary 7*), the rate of evolution of BJEU06-1-Like PRRSV was approximately 4.79 × 10^−3^ substitutions/site/year (95% HPD intervals: 3.32 × 10^−3^–6.19 × 10^−3^). The tMRCA of BJEU06-1-LikePRRSV was estimated to be approximately 1,995.33 (95% HPD range was 1,988.45–2,000.53) (*Supplementary 6*).

## 4. Discussion

PRRSV-1 has been researched in China for more than two decades, and various types of recombinant and pathogenic strains have been reported [9, 10, 11, 13, 14, 15, 16, 17]. With the continuous increase in the total quantity and geographical distribution of Chinese PRRSV-1, studying the complete genome characteristics of the various PRRSV-1 strains is important. In this study, 24 complete genomes from different pig farms were assembled. Subsequently, reference PRRSV-1 sequences from the Chinese strains were retrieved from the NCBI database. Finally, an in-depth analysis of these sequences was performed to elucidate the current genome characteristics of Chinese PRRSV-1. In a previous study, the PRRSV-1 strains from China were classified into four main subgroups [9]. In this study, we revealed that the current PRRSV-1 strains from China belong to Western European Subtype I and demonstrated that PRRSV-1 strains from China can be divided into seven subgroups. This indicates that the genetic diversity of PRRSV-1 in China is increasing. The 24 strains were classified into four different subgroups. The phylogenetic analysis of the complete genomes revealed that BJEU06-1-Like PRRSV accounted for 79.17% of the 24 strains. According to the ORF5 phylogenetic analysis, BJEU06-1-Like PRRSV accounted for 70.83% of the 24 strains. This phenomenon suggests that BJEU06-1-Like PRRSV has become the predominant strain in China. In this study, PRRSV-1 strains from China collected from different pig farms had low pairwise complete genome similarity, mainly ranging from 80% to 90%. The 24 strains had low nucleotide and amino acid sequence similarity with the reference strains from the seven subgroups, especially in ORF1a, Nsp1*β*, and Nsp2. Previous studies have shown that there is a high complete genome similarity of PRRSV-1 strains from China in the same pig farm or same swine herd [25, 45]. We speculated that the reasons for this phenomenon may be as follows: (ⅰ) PRRSV-1 may have undergone long-term evolution in China, and (ⅱ) PRRSV-1 may have been introduced into China multiple times from different regions. However, the evolutionary relationship of PRRSV-1 among different pig farms requires additional research. In this study, we found that there were extensive recombination events in Chinese PRRSV-1, and the recombination events mainly originated from BJEU06-1-Like and NMEU09-1-Like strains. A previous study showed that pervasive recombination events might alter the viral pathogenicity and give rise to viral evolution [46], and pathogenic recombinant strains and virulence-enhancing strains of PRRSV-1 in China have been reported [9, 15]. However, more research is needed to determine whether the enhanced virulence of PRRSV-1 is associated with recombination.

Compared with the prototypic strains Lelystad virus and ATCC VR-2332, indel is the most common phenomenon in the Nsp2 or ORF3 hypervariable regions for many PRRSVs [47, 48, 49, 50, 51]. For PRRSV-2, the predominant circulating strains (HP-PRRSV, NADC30-Like PRRSV, and NADC34-Like PRRSV) exhibited consistent insertions or deletions in the Nsp2 region. The unique molecular hallmark of HP-PRRSV is a discontinuous 30-aa deletion (1 + 29) in Nsp2 [48, 52]. NADC30-Like PRRSV is characterized by discontinuous 131-aa deletions (111 + 1 + 19) in Nsp2 [53, 54, 55]. The consistent deletion feature of NADC34-Like PRRSV is a continuous 100-aa deletion in Nsp2 [56, 57]. For the PRRSV-1 strains from China, four subgroups exhibited regular deletions or insertions in the hypervariable region of the Nsp2 region. BJEU06-1-Like PRRSV strains possessed a discontinuous aa deletion pattern (4 + 1). The remaining subgroups with regular deletions or insertions were new subgroup1, new subgroup 2, and the HKEU16-Like subgroup. However, due to the limited number of strains in these three subgroups, more research is needed to determine whether their Nsp2 exhibits consistent insertion or deletion features. Given the characteristic deletions or insertions observed in the Nsp2 hypervariable region of these four subgroups, they may become predominant epidemic strains in the future. Previous studies have reported that GP3 and GP4 are the most variable structural proteins, especially in the overlapping regions of GP3 and GP4 [9, 20, 58]. In this study, we found that the mutations in the overlapping region mainly included deletions and C-terminal truncation. However, there were no regular deletions or insertions in any of the subgroups. Notably, many PRRSV-1 strains from China exhibited various deletions or premature terminations, which is consistent with previously reported PRRSV-1 strains from other countries [59]. These findings suggest that some PRRSV-1 strains from China may originate from abroad. Previous studies have also speculated on the origin of PRRSV-1 strains from China from multiple perspectives [9, 17, 22, 60]. However, direct evidence of how PRRSV-1 was introduced into China has not yet been found.

Most studies choose the ORF5 gene to predict positively selected codons and evaluate the rate of evolution of PRRSV [61, 62]. In this study, we identified 15 codons under positive selection by five different approaches in site model that were distributed in different regions of GP5, including the signal peptide (SP), putative ectodomain 1 (EcD1), and putative ectodomain 2 (EcD2). One positively selected site (35) in EcD1 can be found in the previously reported neutralization epitope [63]. We speculate that the site may have undergone adaptation under selection pressure from the pig population. Compared with other positively selected sites, three codons (56, 104, and 106) had more combinations of amino acid physicochemical properties. This phenomenon was more pronounced for BJEU06-1-Like PRRSV. Thus, the branch-site model was performed for the seven subgroups. We found that only the BJEU06-1-Like subgroup had statistically significant LRTs. These findings suggest that the BJEU06-1-Like subgroup is under stronger selection than the other subgroups. To investigate the rate of evolution and tMRCA of Chinese PRRSV-1, time-scaled phylogenetic trees of the ORF5 gene were constructed with the BEAST package. The tMRCA of PRRSV-1 strains from China was approximately 1,969.63. Previous studies have suggested that the introduction of PRRSV-1 into China involves four stages corresponding to four types of PRRSV-1 (Amervac-Like, BJEU06-1-Like, HKEU16-Like, and NMEU09-1-Like), indicating that the common ancestor of PRRSV-1 strains from China and the above four types of PRRSV-1 may have already emerged in other countries [17]. In recent years, the detection rate for BJEU06-1-Like PRRSV has rapidly increased, yet its rate of evolution is slightly greater than that of Chinese PRRSV-1. The reasons for this phenomenon may be as follows: (ⅰ) the ORF5 sequence of BJEU06-1-Like PRRSV accounts for a large proportion (approximately 46.67%) of sets, and (ⅱ) there are more mutation types in the key sites of the membrane protein ectodomain for BJEU06-1-Like PRRSV, such as the GP5 protein. Furthermore, when compared with other PRRSVs circulating in a certain swine farm, both PRRSV-1 strains from China and BJEU06-1-Like PRRSV have a lower rate of evolution [25, 64, 65]. The reasons for this phenomenon may include the following: (ⅰ) fewer transmission barriers to PRRSV strains within the same pig farm and (ⅱ) greater and more uniform selection pressure of drugs and pig populations on the same pig farm.

## 5. Conclusion

In conclusion, the genetic diversity of PRRSV-1 strains from China has increased, mainly manifesting in the expansion of the Chinese PRRSV-1 subgroup and the significant differences in the complete genome among PRRSV-1 strains from China from different pig farms. BJEU06-1-Like PRRSV which possesses five discontinued aa deletions in Nsp2 region has been the predominant strains now, and other three Chinese PRRSV-1 subgroups which carry regular deletions or insertions in Nsp2 region also have the potential to become predominant epidemic strains in the future. BJEU06-1-Like and NMEU09-1-Like strains have extensively participated in recent recombination events. PRRSV-1 strains from China possesses a high mutation rate, and the analysis of positive selection indicated that BJEU06-1-Like PRRSV possessed more combinations of amino acid physicochemical properties in the positively selected sites of the GP5 ectodomain than other PRRSV-1 strains from China.

## Figures and Tables

**Table 1:** Information on the recombination events of Chinese PRRSV-1.

Recombinant virus	Classification^a^	Parental virus		Breakpoint			Reference
		Major	Minor	Region	Begin	End	
GDXNF41-1801	BJEU06-1-Like	BJEU06-1	NMEU09-1	5′UTR-nsp1*β*	86	899	This study

HLJZD25-1810	BJEU06-1-Like	BJEU06-1	NMEU09-1	nsp1*α*-nsp1*β*	329	960	This study

GDXNF85-1803	NMEU09-1-Like	LNEU12	NMEU09-1	ORF2a	11,924	12,316	This study
				ORF3-ORF6	12,959	14,440	This study

GDXNF73-1802	NMEU09-1-Like	LNEU12	NMEU09-1	nsp12-ORF6	11,760	14,532	This study

NVDC-NM2	NMEU09-1-Like	NMEU09-1	BJEU06-1	nsp1*β*-nsp2	917	3,416	(Sun et al., 2023)
NVDC-NM3	NMEU09-1-Like	NMEU09-1	BJEU06-1	nsp1*β*-nsp2	917	3,416	
NVDC-FJ	NMEU09-1-Like	NMEU09-1	BJEU06-1	nsp1*β*-nsp2	917	3,416	

HLJB1	Amervac-Like	Amervac PRRS	BJEU06-1	nsp2	3,083	3,732	(Chen et al. [9])
				nsp7*α*-nsp9	6,561	7,730	
				nsp10-ORF3	10,595	12,781	

HeB47	Amervac-Like	BJEU06–1	CReSA228	nsp2-nsp3	3,759	5,209	(Yu et al. [17])
				nsp9-nsp10	8,460	9,340	
				ORF3-3′UTR	12,906	14,992	

TZJ2134	DV + Amervac-like	Amervac PRRS	DV	nsp10-nsp11	9,397	11,266	(Sun et al. [16])

HKEU16	HKEU16-Like	HK5	HK10	5′UTR-nsp2	0	2,108	—
				nsp2	2,341	2,701	
				nsp10	9,621	10,506	
				ORF2-3′UTR	11,982	15,074	
		HK5	Lelystadvirus	nsp9-nsp10	9,185	9,620	

^a^The classification of recombinant PRRSV was based on the phylogenetic tree constructed using ORF5 sequences.

**Table 2:** ORF5 parameter estimates and likelihood ratio test (LRT) for the branch-site model.

Branch-site model A		LRT		*p* Value	Positively selected sites^c^
Foreground branches	Parameter estimates	2*Δ*ℓ^a^	df^b^		
Amervac-Like	*p*_0_ = 0.778, *p*_1_ = 0.222, *p*_2a_ = 0.000, *p*_2b_ = 0.000, *ω*_0_ = 0.079, *ω*_1_ = 1.000, *ω*_2_ = 1.000	0.000	1	n.s.	None
BJEU06-1-Like	*p*_0_ = 0.731, *p*_1_ = 0.244, *p*_2a_ = 0.019, *p*_2b_ = 0.006, *ω*_0_ = 0.075, *ω*_1_ = 1.000, *ω*_2_ = 5.987	87.611	1	0.001	56 ^*∗∗*^, 60 ^*∗∗*^, 61 ^*∗*^, 104 ^*∗∗*^, 106 ^*∗∗*^
HKEU16-Like	*p*_0_ = 0.778, *p*_1_ = 0.222, *p*_2a_ = 0.000, *p*_2b_ = 0.000, *ω*_0_ = 0.079, *ω*_1_ = 1.000, *ω*_2_ = 1.000	0.000	1	n.s.	None
NMEU09-1-Like	*p*_0_ = 0.759, *p*_1_ = 0.214, *p*_2a_ = 0.021, *p*_2b_ = 0.006, *ω*_0_ = 0.077, *ω*_1_ = 1.000, *ω*_2_ = 1.000	0.000	1	n.s.	None
New subgroup 1	*p*_0_ = 0.755, *p*_1_ = 0.215, *p*_2a_ = 0.023, *p*_2b_ = 0.006, *ω*_0_ = 0.079, *ω*_1_ = 1.000, *ω*_2_ = 1.000	0.000	1	n.s.	None
New subgroup 2	*p*_0_ = 0.731, *p*_1_ = 0.211, *p*_2a_ = 0.045, *p*_2b_ = 0.013, *ω*_0_ = 0.076, *ω*_1_ = 1.000, *ω*_2_ = 1.574	0.113	1	n.s.	None
New subgroup 3	*p*_0_ = 0.778, *p*_1_ = 0.222, *p*_2a_ = 0.000, *p*_2b_ = 0.000, *ω*_0_ = 0.079, *ω*_1_ = 1.000, *ω*_2_ = 1.000	0.000	1	n.s.	None

^a^2*Δ*ℓ, likelihood ratio test (LRT) to identify positive selection. ^b^df, degrees of freedom. ^c^Positively selected sites which have probability higher than 95% ( ^*∗*^) or 99% ( ^*∗∗*^) in the Bayes empirical Bayes (BEB) analyses.

## Data Availability

The sequences of this study were deposited in GenBank with the accession number PP330948-PP330950, PP336343-PP336346, PP341288-PP341290, PP350850-PP350855, and PP402109-PP402114. The sequences will be released to public databases when the data or accession number appear in print. The sequences data are supplied in the supplementary files.

## Supplementary Materials

Supplementary 1 Table 1: nucleotide similarity of PRRSV-1 strains from China between the four subgroups in this study and seven reference subgroups.

Supplementary 2 Table 2: the amino acid similarity of PRRSV-1 strains from China between the four subgroups in this study and seven reference subgroups.

Supplementary 3 Figure 1: amino acid alignment of GP3 (a) and GP4 (b).

Supplementary 4 Figure 2: recombination analysis of strains GDXNF41-1801, HJLZD25-1810, GDXNF73-1802, GDXNF85-1803, and HKEU16.

Supplementary 5 Table 3: marginal likelihoods estimated by molecular clock models and coalescent models for Chinese PRRSV-1.

Supplementary 6 Figure 3: estimation of the median time to the most recent common ancestor (tMRCA) for PRRSV-1 strains from China based on the ORF5 gene.

Supplementary 7 Table 4: marginal likelihoods estimated by molecular clock models and coalescent models for BJEU06-1-Like PRRSV.
