# Supplementary material for: Dissecting Genetic Diversity and Evolutionary Trends of Chinese PRRSV-1 Based on Whole-Genome Analysis
Source: Transbound Emerg Dis. 2024 Jun 11;2024:9705539. doi: 10.1155/2024/9705539 (PMC12017348; doi:10.1155/2024/9705539)
Supplement: Supplementary 1 — Table 1: nucleotide similarity of PRRSV-1 strains from China between the four subgroups in this study and seven reference subgroups. [file 9705539.f1.docx]

**Table S1 Nucleotide similarity of PRRSV-1 strains from China between the four subgroups in this study and seven reference subgroups**

| Genes | Subgroups/strains | ATCC VR-2332 | Lelystad virus | Amervac-Like | BJEU06-1-Like | HKEU16-Like | NMEU09-1-Like | New subgroup 1 | New subgroup 2 | New subgroup 3 |
| --- | --- | --- | --- | --- | --- | --- | --- | --- | --- | --- |
| 3UTR | Amervac-Like^a^ BJEU06-1-Like^b^ NMEU09-1-Like^c^ New subgroup 2^d^ | 74.8~75.2 74.3~77.9 74.3~80.5 76.1~76.1 | 89.3~92.2 91.4~95.7 87.9~94.0 95.6~95.6 | 88.4~94.8 87.1~96.5 85.3~95.6 91.2~95.6 | 84.7~89.7 90.1~99.1 86.0~98.3 91.9~98.2 | 87.4~93.0 87.4~93.7 85.3~93.7 92.6~95.8 | 82.1~91.2 87.1~96.5 88.7~97.4 91.2~97.4 | 88.4~91.2 87.7~93.6 87.7~92.1 92.1~92.1 | 87.5~90.5 92.2~96.6 89.7~95.7 97.4~97.4 | 89.2~92.0 91.2~94.7 91.2~93.8 92.9~92.9 |
| 5UTR | Amervac-Like BJEU06-1-Like NMEU09-1-Like New subgroup 2 | 60.6~61.2 59.6~62.8 59.6~62.2 58.8~58.8 | 94.1~94.1 92.8~97.3 94.6~95.9 95.5~95.5 | 91.4~94.1 91.9~98.2 92.8~97.7 93.2~95.9 | 86.4~95.5 84.6~99.1 85.0~98.6 87.7~94.5 | 93.2~99.1 91.4~100.0 92.7~98.1 94.1~94.4 | 91.9~93.2 91.0~97.3 93.2~96.8 93.2~94.5 | 91.4~92.3 90.5~95.5 93.2~95.0 92.7~92.7 | 91.4~91.9 89.5~95.7 92.4~93.8 94.3~94.3 | 91.0~91.9 89.6~93.2 90.0~93.7 94.1~94.1 |
| ORF1a | Amervac-Like BJEU06-1-Like NMEU09-1-Like New subgroup 2 | 55.2~55.5 55.1~56.6 55.1~55.9 55.4~55.4 | 83.2~83.3 85.1~87.1 82.4~86.7 85.4~85.4 | 80.0~82.1 81.6~88.4 79.9~88.0 81.5~83.5 | 78.8~80.8 80.9~96.9 78.3~91.0 80.0~82.3 | 81.1~81.9 81.8~83.5 79.8~83.6 82.1~82.2 | 77.9~79.2 78.8~85.3 79.0~93.3 79.2~80.1 | 80.4~80.6 79.4~81.2 78.6~80.1 79.3~79.3 | 78.6~78.6 79.7~80.7 78.4~80.4 89.9~89.9 | 78.3~78.7 79.2~80.5 78.3~80.1 79.3~79.3 |
| ORF1b | Amervac-Like BJEU06-1-Like NMEU09-1-Like New subgroup 2 | 63.0~63.1 62.8~63.6 62.9~63.3 63.4~63.4 | 87.8~87.8 88.0~90.9 86.4~90.5 88.9~88.9 | 85.7~87.3 86.3~91.5 84.7~91.0 86.4~88.0 | 84.3~85.9 84.4~97.7 83.5~93.2 85.1~86.3 | 85.5~86.1 85.2~87.8 84.9~88.2 86.2~87.0 | 83.1~83.9 83.6~85.9 84.0~94.8 83.3~84.8 | 84.5~85.0 83.7~84.8 83.1~85.2 84.3~84.3 | 84.3~84.6 83.7~85.6 83.1~85.1 93.6~93.6 | 82.8~82.8 82.4~83.9 82.4~83.6 83.2~83.2 |
| ORF2a | Amervac-Like BJEU06-1-Like NMEU09-1-Like New subgroup 2 | 65.1~65.6 63.5~67.1 65.5~66.0 64.1~64.1 | 87.1~87.6 86.9~92.7 87.5~89.3 88.8~88.8 | 84.1~86.5 85.1~94.3 85.3~90.0 86.1~87.7 | 82.5~86.4 85.5~97.9 84.7~88.1 84.7~87.3 | 84.7~87.1 85.5~90.0 86.7~90.9 85.6~87.7 | 83.5~85.7 83.9~87.5 87.7~95.9 85.3~85.9 | 83.3~83.7 83.9~87.2 85.1~86.0 85.7~85.7 | 83.7~84.5 84.9~87.3 84.8~86.5 94.9~94.9 | 83.5~83.5 83.5~85.9 83.5~85.6 84.0~84.0 |
| ORF2b | Amervac-Like BJEU06-1-Like NMEU09-1-Like New subgroup 2 | 71.4~71.8 72.3~75.1 74.6~75.6 72.8~72.8 | 91.5~92.0 91.5~95.3 92.5~95.3 93.9~93.9 | 87.8~92.0 89.2~98.1 88.7~94.8 88.7~94.4 | 87.3~92.0 90.1~98.6 88.7~93.0 89.2~93.4 | 86.9~90.6 86.9~93.9 86.9~94.4 87.8~93.4 | 88.3~92.0 87.3~94.8 89.2~98.6 89.2~92.5 | 87.3~89.7 88.3~93.0 90.1~92.0 91.1~91.1 | 91.5~91.5 90.1~94.8 91.1~95.3 96.7~96.7 | 87.3~87.3 87.3~90.1 88.7~90.1 88.7~88.7 |

Table S1 (continued)

| Genes | Subgroups/strains | ATCC VR-2332 | Lelystad virus | Amervac-Like | BJEU06-1-Like | HKEU16-Like | NMEU09-1-Like | New subgroup 1 | New subgroup 2 | New subgroup 3 |
| --- | --- | --- | --- | --- | --- | --- | --- | --- | --- | --- |
| ORF3 | Amervac-Like BJEU06-1-Like NMEU09-1-Like New subgroup 2 | 65.0~66.0 63.9~66.4 64.5~67.2 66.0~66.0 | 83.8~84.0 85.1~90.6 86.7~90.8 85.8~85.8 | 81.3~83.9 82.3~91.2 84.0~91.6 83.2~85.1 | 79.3~83.3 81.8~96.4 82.9~89.8 81.2~84.6 | 81.6~84.1 82.4~87.9 82.8~88.1 85.4~87.1 | 80.0~82.5 81.4~87.2 86.2~94.3 84.5~86.9 | 83.0~83.3 80.8~85.2 83.8~87.0 83.8~83.8 | 83.0~84.5 83.1~85.1 84.2~85.9 92.0~92.0 | 81.5~82.6 80.3~85.7 83.6~84.2 81.6~81.6 |
| ORF4 | Amervac-Like BJEU06-1-Like NMEU09-1-Like New subgroup 2 | 67.8~68.0 65.8~68.5 65.1~67.9 68.3~68.3 | 84.2~85.5 84.9~88.8 87.1~89.2 86.7~86.7 | 83.2~85.7 82.1~88.6 85.4~90.0 84.1~86.3 | 80.0~85.4 83.7~98.0 83.1~90.3 80.9~85.7 | 82.2~84.3 83.1~87.5 85.6~90.9 84.6~88.8 | 81.4~85.2 82.4~88.3 86.6~96.0 85.6~88.0 | 84.7~84.9 81.7~84.7 83.5~85.4 83.9~83.9 | 82.2~83.1 80.7~85.7 83.3~84.1 87.0~87.0 | 83.6~83.6 81.2~85.5 84.7~86.0 82.6~82.6 |
| ORF5 | Amervac-Like BJEU06-1-Like NMEU09-1-Like New subgroup 2 | 64.3~64.8 61.3~62.9 61.1~63.5 64.1~64.1 | 86.6~89.6 84.0~88.0 82.2~86.8 85.5~85.5 | 86.5~89.1 83.3~90.4 80.5~88.1 83.7~86.3 | 82.2~85.8 84.8~96.9 79.9~85.8 82.3~86.3 | 82.8~87.0 82.7~87.0 80.4~86.5 83.2~84.8 | 81.4~83.7 80.5~86.0 85.3~92.2 82.0~84.0 | 83.0~85.5 80.5~84.2 78.2~83.7 82.3~82.3 | 83.7~85.1 83.7~86.8 81.2~84.5 89.4~89.4 | 80.4~80.9 81.2~84.3 79.0~82.5 83.0~83.0 |
| ORF5a | Amervac-Like BJEU06-1-Like NMEU09-1-Like New subgroup 2 | 59.1~59.8 58.3~62.1 58.3~59.8 61.4~61.4 | 95.5~97.0 90.9~96.2 87.1~93.2 91.7~91.7 | 90.2~94.7 88.6~97.0 82.6~93.2 87.9~91.7 | 89.4~97.0 87.1~99.2 81.1~93.2 87.1~92.4 | 93.9~97.0 87.9~97.0 85.6~93.2 91.7~93.2 | 88.6~96.2 85.6~95.5 86.4~97.0 86.4~92.4 | 87.9~89.4 85.6~93.2 84.8~87.9 85.6~85.6 | 94.7~94.7 86.4~94.7 83.3~88.6 88.6~88.6 | 83.3~83.3 78.0~84.1 78.0~81.1 81.1~81.1 |
| ORF6 | Amervac-Like BJEU06-1-Like NMEU09-1-Like New subgroup 2 | 68.0~69.9 68.2~69.9 68.4~70.1 67.8~67.8 | 90.0~91.4 90.4~93.3 88.1~90.8 88.9~88.9 | 87.9~90.6 87.5~93.7 87.4~92.0 88.1~89.8 | 87.4~90.0 88.9~98.9 85.6~90.6 86.4~88.3 | 87.0~87.7 87.2~90.4 88.5~90.8 89.7~90.2 | 85.6~89.3 86.4~90.6 88.5~96.0 87.0~88.7 | 85.6~86.0 87.2~88.9 85.6~88.5 87.4~87.4 | 87.4~87.7 87.0~89.1 88.3~90.2 88.1~88.1 | 86.8~87.4 85.6~88.7 84.5~86.4 85.6~85.6 |
| ORF7 | Amervac-Like BJEU06-1-Like NMEU09-1-Like New subgroup 2 | 65.8~66.7 64.7~69.2 65.6~67.2 67.8~67.8 | 89.7~91.5 91.2~93.8 92.0~94.1 93.8~93.8 | 88.9~92.0 88.4~93.5 88.4~93.0 89.7~91.5 | 88.1~91.0 89.7~97.9 87.3~95.2 88.6~90.7 | 85.5~88.4 86.3~91.0 87.3~91.7 89.1~89.7 | 85.8~90.2 86.0~91.2 86.3~95.3 89.1~91.7 | 87.3~89.7 87.3~89.7 87.3~89.9 88.1~88.1 | 86.3~88.9 88.4~90.2 88.9~90.7 94.8~94.8 | 84.5~85.5 84.2~87.6 85.8~88.4 87.1~87.1 |

Table S1 (continued)

| Genes | Subgroups/strains | ATCC VR-2332 | Lelystad virus | Amervac-Like | BJEU06-1-Like | HKEU16-Like | NMEU09-1-Like | New subgroup 1 | New subgroup 2 | New subgroup 3 |
| --- | --- | --- | --- | --- | --- | --- | --- | --- | --- | --- |
| Genome | Amervac-Like BJEU06-1-Like NMEU09-1-Like New subgroup 2 | 60.3~60.3 59.9~60.9 60.0~60.3 60.2~60.2 | 85.6~86.0 86.7~88.9 84.9~88.5 87.2~87.2 | 83.3~85.1 84.2~90.0 83.1~89.4 84.1~85.7 | 82.0~83.8 83.6~97.3 81.5~91.1 82.9~84.5 | 83.4~84.0 83.9~85.5 83.0~85.6 84.5~84.7 | 81.4~81.9 82.1~85.2 82.9~94.2 82.5~83.1 | 82.8~82.9 82.1~83.1 81.7~83.0 82.2~82.2 | 81.8~82.1 82.4~83.3 81.7~83.2 91.4~91.4 | 81.0~81.1 81.2~82.3 80.9~82.3 81.6~81.6 |

a. Amervac-Like PRRSVs in this study (PY61 and TZJ2780).

b. BJEU06-1-Like PRRSVs in this study (ZD-1, TZJ637, GDXNF41-1801, HLJTZJ155-2001, HLJWG9-1612, HLJWK14-1611, HLJWK335-2005, HLJZD25-1810, HNLCL53-1812, HNLCL7-1804, HNLCL75-1812, IMWK141-1801, LNDB50-1806, TJWK169-1804, XJTZJ158-2001, TZJ2781, and ZZH817).

c. NMEU09-1-Like PRRSVs in this study (GDXNF161-1806, GDXNF73-1802, GDXNF85-1803, and GDXNF94-1804).

d. New subgroup 2 strain in this study (SDHSW160-2201).
