# Supplementary material for: Dissecting Genetic Diversity and Evolutionary Trends of Chinese PRRSV-1 Based on Whole-Genome Analysis
Source: Transbound Emerg Dis. 2024 Jun 11;2024:9705539. doi: 10.1155/2024/9705539 (PMC12017348; doi:10.1155/2024/9705539)
Supplement: Supplementary 2 — Table 2: the amino acid similarity of PRRSV-1 strains from China between the four subgroups in this study and seven reference subgroups. [file 9705539.f2.docx]

**Table S2 The amino acid similarity of PRRSV-1 strains from China between the four subgroups in this study and seven reference subgroups**

| Proteins | Subgroups/strains | ATCC VR-2332 | Lelystad virus | Amervac-Like | BJEU06-1-Like | HKEU16-Like | NMEU09-1-Like | New subgroup 1 | New subgroup 2 | New subgroup 3 |
| --- | --- | --- | --- | --- | --- | --- | --- | --- | --- | --- |
| Nsp1a | Amervac-Like^a^ BJEU06-1-Like^b^ NMEU09-1-Like^c^ New subgroup 2^d^ | 65.6~67.8 64.4~67.8 65.6~66.7 65.0~65.0 | 88.3~88.9 90.0~93.9 91.1~94.4 94.4~94.4 | 87.2~89.4 87.8~96.7 89.4~95.0 89.4~92.2 | 85.6~88.9 87.8~97.8 88.3~95.6 88.3~91.7 | 86.7~90.6 86.7~92.8 88.3~92.2 88.3~89.4 | 85.0~91.7 85.6~95.6 88.9~95.6 88.9~92.8 | 87.2~88.3 88.9~92.2 90.6~91.7 90.6~90.6 | 87.2~87.2 87.8~92.2 87.8~91.7 92.2~92.2 | 88.9~90.6 88.9~93.3 90.0~92.8 91.7~91.7 |
| Nsp1b | Amervac-Like BJEU06-1-Like NMEU09-1-Like New subgroup 2 | 42.4~43.4 42.4~45.5 42.9~45.5 42.9~42.9 | 77.1~78.5 77.6~82.9 77.6~83.9 80.5~80.5 | 73.7~78.5 75.6~90.2 72.7~90.2 77.6~80.0 | 72.7~77.6 75.6~93.7 75.1~89.8 76.1~79.5 | 75.1~81.5 74.6~81.0 76.1~82.4 75.6~78.5 | 73.2~76.6 72.7~86.3 75.6~90.2 74.1~75.6 | 68.8~70.2 70.7~81.0 69.3~74.1 71.7~71.7 | 71.2~73.7 73.7~78.0 73.2~76.6 82.0~82.0 | 76.6~76.6 75.6~79.5 75.1~81.5 76.6~76.6 |
| Nsp2 | Amervac-Like BJEU06-1-Like NMEU09-1-Like New subgroup 2 | 38.2~38.6 38.4~39.9 38.4~40.4 38.6~38.6 | 80.8~81.3 79.7~84.3 76.6~82.4 82.1~82.1 | 75.7~79.6 77.0~85.1 74.9~84.7 76.0~79.4 | 74.7~77.8 77.2~96.4 72.1~87.4 74.8~78.0 | 75.9~77.9 74.7~80.1 72.9~79.8 76.8~77.9 | 71.6~77.0 70.8~85.4 74.0~90.4 73.2~75.6 | 76.3~77.7 73.1~75.1 71.2~74.2 74.6~74.6 | 73.5~74.0 72.5~75.0 70.5~75.2 87.2~87.2 | 73.0~73.1 71.6~74.7 71.7~74.0 73.2~73.2 |
| Nsp3 | Amervac-Like BJEU06-1-Like NMEU09-1-Like New subgroup 2 | 51.3~52.6 50.4~52.6 50.4~52.2 51.7~51.7 | 93.0~94.8 92.6~95.7 91.7~95.2 93.0~93.0 | 92.2~94.8 91.3~95.7 92.2~96.1 92.6~93.9 | 91.3~93.5 92.2~99.6 91.7~98.3 90.9~92.6 | 91.7~94.3 90.0~95.7 90.9~96.5 92.2~93.0 | 91.7~93.5 90.0~95.7 91.7~97.0 91.7~93.5 | \^e^ | 93.9~94.3 91.3~94.8 92.2~93.9 95.2~95.2 | 91.7~91.7 91.3~94.8 90.9~93.0 90.9~90.9 |
| Nsp4 | Amervac-Like BJEU06-1-Like NMEU09-1-Like New subgroup 2 | 60.6~61.1 58.6~61.1 60.1~61.6 60.1~60.1 | 90.1~92.1 88.7~94.1 88.7~91.6 91.6~91.6 | 89.7~93.1 88.7~94.1 86.7~93.6 91.1~92.6 | 86.7~91.1 86.2~98.5 84.2~97.0 85.7~89.7 | 88.7~92.6 86.7~94.1 85.7~91.1 90.1~91.6 | 85.2~88.2 82.3~89.7 83.3~96.6 84.7~88.2 | 91.6~92.1 86.2~90.6 85.2~87.2 89.2~89.2 | 90.1~90.1 86.2~91.6 87.2~91.6 95.1~95.1 | 91.6~92.1 87.2~93.1 87.2~89.7 89.2~89.2 |
| Nsp5 | Amervac-Like BJEU06-1-Like NMEU09-1-Like New subgroup 2 | 66.5~67.1 65.9~69.4 66.5~67.1 66.5~66.5 | 87.1~91.8 91.2~96.5 90.0~93.5 91.2~91.2 | 84.7~95.9 88.8~97.1 89.4~94.1 89.4~91.8 | 84.7~92.4 85.3~99.4 84.7~97.6 90.6~92.9 | 87.1~91.8 87.1~98.2 90.0~94.1 91.8~92.9 | 85.9~88.2 85.9~92.4 85.9~97.6 88.2~91.2 | 90.0~90.6 88.2~92.9 88.8~90.6 88.8~88.8 | 87.1~89.4 87.6~94.1 88.8~92.9 95.3~95.3 | 87.6~89.4 88.2~95.3 88.2~91.8 90.6~90.6 |

**Table S2 (continued)**

| Proteins | Subgroups/strains | ATCC VR-2332 | Lelystad virus | Amervac-Like | BJEU06-1-Like | HKEU16-Like | NMEU09-1-Like | New subgroup 1 | New subgroup 2 | New subgroup 3 |
| --- | --- | --- | --- | --- | --- | --- | --- | --- | --- | --- |
| Nsp6 | Amervac-Like BJEU06-1-Like NMEU09-1-Like New subgroup 2 | 75.0~81.2 75.0~87.5 81.2~81.2 75.0~75.0 | 93.8~100.0 87.5~100.0 100.0~100.0 93.8~93.8 | 93.8~100.0 87.5~100.0 100.0~100.0 93.8~93.8 | 87.5~100.0 81.2~100.0 93.8~100.0 87.5~100.0 | 93.8~100.0 81.2~100.0 93.8~100.0 93.8~100.0 | 93.8~100.0 87.5~100.0 100.0~100.0 93.8~93.8 | 87.5~93.8 81.2~93.8 93.8~93.8 87.5~87.5 | 93.8~100.0 81.2~100.0 93.8~93.8 100.0~100.0 | 87.5~93.8 81.2~93.8 93.8~93.8 87.5~87.5 |
| Nsp7a | Amervac-Like BJEU06-1-Like NMEU09-1-Like New subgroup 2 | 59.1~60.4 55.7~60.4 57.7~59.1 58.4~58.4 | 94.0~95.3 92.6~96.6 92.6~95.3 97.3~97.3 | 91.9~96.0 90.6~97.3 90.6~96.0 93.3~96.0 | 91.3~96.6 91.3~98.7 89.3~99.3 92.6~96.0 | 92.6~94.6 91.3~96.6 90.6~96.0 94.0~95.3 | 91.3~95.3 89.3~96.6 90.6~96.6 93.3~96.6 | 95.3~96.0 91.9~95.3 91.9~94.0 95.3~95.3 | 94.6~95.3 92.6~96.6 93.3~95.3 97.3~97.3 | 94.0~94.6 90.6~96.0 92.6~93.3 96.0~96.0 |
| Nsp7b | Amervac-Like BJEU06-1-Like NMEU09-1-Like New subgroup 2 | 36.2~36.2 31.4~36.2 33.3~35.2 34.3~34.3 | 92.5~93.3 89.2~95.0 87.5~93.3 95.0~95.0 | 89.2~91.7 87.5~95.8 86.7~95.0 92.5~94.2 | 86.7~92.5 85.8~98.3 85.8~95.8 89.2~95.0 | 90.0~92.5 88.3~95.0 85.0~92.5 94.2~95.0 | 85.0~88.3 84.2~92.5 87.5~93.3 88.3~91.7 | 85.8~88.3 81.7~87.5 82.5~86.7 86.7~86.7 | 89.2~90.8 85.0~91.7 85.8~91.7 93.3~93.3 | 83.3~85.0 81.7~88.3 85.0~86.7 87.5~87.5 |
| Nsp8 | Amervac-Like BJEU06-1-Like NMEU09-1-Like New subgroup 2 | 64.4~64.4 62.2~71.1 64.4~66.7 66.7~66.7 | 88.9~91.1 88.9~97.8 93.3~95.6 93.3~93.3 | 84.4~93.3 84.4~100.0 88.9~95.6 91.1~93.3 | 77.8~88.9 84.4~100.0 86.7~95.6 82.2~91.1 | 86.7~93.3 84.4~97.8 86.7~97.8 91.1~95.6 | 86.7~91.1 86.7~97.8 91.1~100.0 91.1~93.3 | \^e^ | 91.1~93.3 86.7~97.8 91.1~97.8 95.6~95.6 | 88.9~91.1 88.9~97.8 93.3~95.6 93.3~93.3 |
| Nsp9 | Amervac-Like BJEU06-1-Like NMEU09-1-Like New subgroup 2 | 73.8~74.1 73.3~75.2 74.4~74.8 74.2~74.2 | 96.1~96.6 95.5~97.8 93.6~96.7 96.3~96.3 | 95.3~96.7 94.6~98.3 94.0~97.1 95.8~96.4 | 94.4~95.3 94.0~98.9 92.2~97.5 94.6~95.7 | 95.3~95.8 94.4~96.6 92.9~95.5 95.7~96.0 | 94.1~94.9 93.5~95.8 93.5~98.6 94.1~94.9 | \^e^ | 95.3~95.3 94.3~95.7 93.5~94.7 97.2~97.2 | 93.8~94.3 93.0~94.9 93.3~94.4 93.8~93.8 |
| Nsp10 | Amervac-Like BJEU06-1-Like NMEU09-1-Like New subgroup 2 | 64.9~64.9 63.5~65.5 63.7~64.6 65.1~65.1 | 95.9~96.8 94.1~96.4 93.2~94.6 93.7~93.7 | 94.6~96.2 93.2~97.1 92.1~95.2 93.2~93.9 | 92.5~95.9 91.6~98.2 91.4~97.1 91.6~94.3 | 92.8~95.2 91.2~95.9 90.7~95.0 91.4~93.9 | 91.9~93.2 90.3~94.3 91.0~98.6 91.0~92.3 | 92.5~92.8 90.5~93.0 90.0~91.6 91.6~91.6 | 91.9~92.8 90.7~93.2 90.5~92.1 96.6~96.6 | 91.6~92.1 90.7~93.4 91.0~92.5 92.5~92.5 |

**Table S2 (continued)**

| Proteins | Subgroups/strains | ATCC VR-2332 | Lelystad virus | Amervac-Like | BJEU06-1-Like | HKEU16-Like | NMEU09-1-Like | New subgroup 1 | New subgroup 2 | New subgroup 3 |
| --- | --- | --- | --- | --- | --- | --- | --- | --- | --- | --- |
| Nsp11 | Amervac-Like BJEU06-1-Like NMEU09-1-Like New subgroup 2 | 75.8~76.2 74.9~76.7 74.9~75.8 75.8~75.8 | 95.5~96.0 96.0~97.3 95.5~97.8 98.2~98.2 | 94.6~96.0 95.1~98.2 94.6~97.8 97.8~98.7 | 92.9~96.4 93.3~99.6 93.8~98.7 93.8~97.8 | 94.2~95.5 95.1~97.3 94.6~97.3 96.0~96.9 | 93.8~96.4 94.2~97.8 94.6~99.1 95.5~97.3 | 95.1~96.0 94.6~96.0 93.8~96.4 95.5~95.5 | 93.8~94.2 94.6~96.4 94.6~96.0 98.2~98.2 | 94.6~95.1 95.1~97.8 95.5~97.3 96.9~96.9 |
| Nsp12 | Amervac-Like BJEU06-1-Like NMEU09-1-Like New subgroup 2 | 42.0~43.4 40.6~44.8 42.0~42.7 43.4~43.4 | 89.5~90.8 88.9~97.4 93.5~96.1 95.4~95.4 | 88.9~90.8 88.9~98.0 92.8~97.4 93.5~96.1 | 87.6~92.8 85.6~99.3 89.5~96.1 92.2~94.8 | 90.2~92.8 87.6~96.1 92.8~96.1 94.8~96.1 | 87.6~89.5 86.3~94.1 91.5~98.0 91.5~95.4 | 90.8~91.5 86.3~91.5 90.2~92.2 92.2~92.2 | 91.5~94.8 88.2~93.5 90.2~94.8 96.7~96.7 | 88.7~89.3 86.7~93.3 90.7~92.7 92.0~92.0 |
| GP2 | Amervac-Like BJEU06-1-Like NMEU09-1-Like New subgroup 2 | 61.6~62.0 59.6~64.4 61.2~64.4 61.6~61.6 | 87.6~90.0 89.6~93.6 89.2~91.6 90.0~90.0 | 82.8~87.6 85.2~96.4 84.8~91.2 86.4~88.0 | 82.4~87.6 88.0~97.2 85.2~91.2 84.4~88.4 | 86.0~89.2 85.6~91.6 86.4~90.4 86.4~89.2 | 85.2~88.4 84.8~90.8 87.6~96.8 86.8~87.6 | 85.6~86.8 85.6~91.2 86.4~88.8 86.0~86.0 | 86.4~87.2 84.8~89.2 85.2~88.8 94.4~94.4 | 85.2~86.8 83.6~88.4 84.4~86.4 85.2~85.2 |
| E | Amervac-Like BJEU06-1-Like NMEU09-1-Like New subgroup 2 | 69.0~71.8 71.8~76.1 76.1~77.5 74.6~74.6 | 90.1~90.1 90.1~97.2 93.0~97.2 94.4~94.4 | 88.7~93.0 88.7~100.0 91.5~100.0 90.1~97.2 | 87.3~93.0 90.1~100.0 88.7~97.2 91.5~95.8 | 85.9~93.0 85.9~97.2 91.5~97.2 90.1~94.4 | 88.7~90.1 88.7~97.2 93.0~100.0 91.5~94.4 | 88.7~90.1 87.3~94.4 90.1~94.4 91.5~91.5 | 88.7~88.7 90.1~95.8 95.8~95.8 98.6~98.6 | 85.9~87.3 87.3~93.0 91.5~93.0 93.0~93.0 |
| GP3 | Amervac-Like BJEU06-1-Like NMEU09-1-Like New subgroup 2 | 58.7~59.0 55.9~62.8 56.3~59.8 57.9~57.9 | 79.3~79.3 81.5~89.5 82.6~87.6 82.8~82.8 | 78.1~80.1 80.1~89.8 79.8~88.0 79.0~81.3 | 75.7~81.2 78.3~98.8 78.8~87.6 80.2~83.9 | 76.7~82.7 79.3~88.1 79.5~86.8 82.4~85.7 | 77.0~81.0 76.7~87.3 84.0~93.4 81.7~85.7 | 80.7~82.4 78.6~86.0 82.6~86.0 85.6~85.6 | 79.4~79.8 79.4~85.8 82.6~84.1 88.9~88.9 | 78.1~78.8 77.4~87.2 81.0~83.3 78.2~78.2 |
| GP4 | Amervac-Like BJEU06-1-Like NMEU09-1-Like New subgroup 2 | 64.8~65.9 65.7~69.9 66.3~68.0 68.8~68.8 | 84.8~85.3 84.8~89.8 89.2~91.5 87.8~87.8 | 83.7~87.0 83.1~90.5 88.1~92.6 84.4~87.8 | 78.7~86.4 82.5~97.8 82.4~91.5 78.3~90.6 | 80.4~86.4 81.8~89.2 85.8~93.8 85.0~88.1 | 84.1~88.6 84.7~92.0 88.0~95.5 85.8~88.1 | 84.7~85.8 81.4~85.8 86.9~87.5 82.2~82.2 | 83.3~84.4 81.7~88.8 84.7~86.4 86.7~86.7 | 85.2~85.8 84.2~89.4 88.6~90.3 84.4~84.4 |

**Table S2 (continued)**

| Proteins | Subgroups/strains | ATCC VR-2332 | Lelystad virus | Amervac-Like | BJEU06-1-Like | HKEU16-Like | NMEU09-1-Like | New subgroup 1 | New subgroup 2 | New subgroup 3 |
| --- | --- | --- | --- | --- | --- | --- | --- | --- | --- | --- |
| GP5 | Amervac-Like BJEU06-1-Like NMEU09-1-Like New subgroup 2 | 55.3~56.3 54.3~57.9 52.3~55.3 56.3~56.3 | 87.1~90.1 85.1~90.1 84.2~88.1 84.7~84.7 | 85.1~92.1 86.1~93.6 82.7~90.6 84.2~87.1 | 81.7~89.6 83.2~95.5 80.7~90.1 82.7~88.6 | 85.6~91.1 85.6~92.1 82.7~89.1 86.6~88.1 | 80.7~85.6 81.7~90.1 86.6~93.1 82.2~85.6 | 84.7~86.1 82.7~87.1 79.2~82.7 82.2~82.2 | 85.6~86.6 84.2~89.1 82.2~86.6 87.6~87.6 | 81.2~83.7 82.2~86.1 81.7~85.1 83.2~83.2 |
| ORF5a | Amervac-Like BJEU06-1-Like NMEU09-1-Like New subgroup 2 | 47.7~52.3 47.7~52.3 50.0~52.3 50.0~50.0 | 93.2~95.5 88.6~95.5 81.8~90.9 95.5~95.5 | 90.9~95.5 86.4~97.7 79.5~93.2 90.9~95.5 | 88.6~97.7 86.4~100.0 77.3~93.2 88.6~95.5 | 90.9~97.7 86.4~97.7 79.5~93.2 93.2~97.7 | 86.4~95.5 79.5~95.5 81.8~97.7 86.4~95.5 | 86.4~86.4 86.4~93.2 79.5~84.1 86.4~86.4 | 88.6~90.9 84.1~90.9 79.5~90.9 90.9~90.9 | 79.5~79.5 79.5~81.8 70.5~79.5 81.8~81.8 |
| M | Amervac-Like BJEU06-1-Like NMEU09-1-Like New subgroup 2 | 74.7~76.4 76.4~79.3 76.4~78.2 77.0~77.0 | 91.4~92.0 92.0~97.1 89.7~92.5 92.0~92.0 | 90.8~92.5 92.5~97.7 89.1~93.7 91.4~93.1 | 87.4~92.5 87.9~100.0 87.4~93.7 88.5~93.1 | 88.5~89.7 88.5~93.7 90.2~91.4 92.0~93.7 | 85.6~90.2 87.9~93.7 89.1~97.1 88.5~92.0 | 89.1~89.1 90.2~93.1 87.4~90.2 90.2~90.2 | 89.7~90.2 89.1~94.3 90.8~93.1 91.4~91.4 | 88.5~89.1 88.5~92.0 87.4~89.7 90.2~90.2 |
| N | Amervac-Like BJEU06-1-Like NMEU09-1-Like New subgroup 2 | 60.3~60.3 61.2~64.5 60.3~62.0 62.0~62.0 | 89.1~91.5 89.9~93.8 90.7~96.9 96.1~96.1 | 86.8~93.8 86.8~96.1 88.4~96.1 89.9~93.8 | 88.4~92.2 89.1~98.4 84.5~95.3 87.6~91.5 | 83.7~88.4 83.7~87.6 83.7~89.9 88.4~89.9 | 84.5~91.5 86.0~94.6 84.5~95.3 90.7~93.0 | 87.6~91.5 87.6~90.7 86.0~91.5 89.9~89.9 | 84.5~88.4 87.6~91.5 87.6~92.2 94.6~94.6 | 82.9~86.8 82.9~88.4 83.7~88.4 88.4~88.4 |

a. Amervac-Like PRRSVs strains in this study (PY61 and TZJ2780).

b. BJEU06-1-Like PRRSVs strains in this study (ZD-1, TZJ637, GDXNF41-1801, HLJTZJ155-2001, HLJWG9-1612, HLJWK14-1611, HLJWK335-2005, HLJZD25-1810, HNLCL53-1812, HNLCL7-1804, HNLCL75-1812, IMWK141-1801, LNDB50-1806, TJWK169-1804, XJTZJ158-2001, TZJ2781, and ZZH817).

c. NMEU09-1-Like PRRSVs strains in this study (GDXNF161-1806, GDXNF73-1802, GDXNF85-1803, and GDXNF94-1804).

d. New subgroup 2 strain in this study (SDHSW160-2201).

e. The New subgroup 1 reference strain (EUGDHD2018) was excluded when performing amino acid alignment of Nsp3, Nsp8 and Nsp9, as these proteins of EUGDHD2018 were false.
