# Supplementary material for: Dissecting Genetic Diversity and Evolutionary Trends of Chinese PRRSV-1 Based on Whole-Genome Analysis
Source: Transbound Emerg Dis. 2024 Jun 11;2024:9705539. doi: 10.1155/2024/9705539 (PMC12017348; doi:10.1155/2024/9705539)
Supplement: Supplementary 3 — Figure 1: amino acid alignment of GP3 (a) and GP4 (b). [file 9705539.f3.docx]

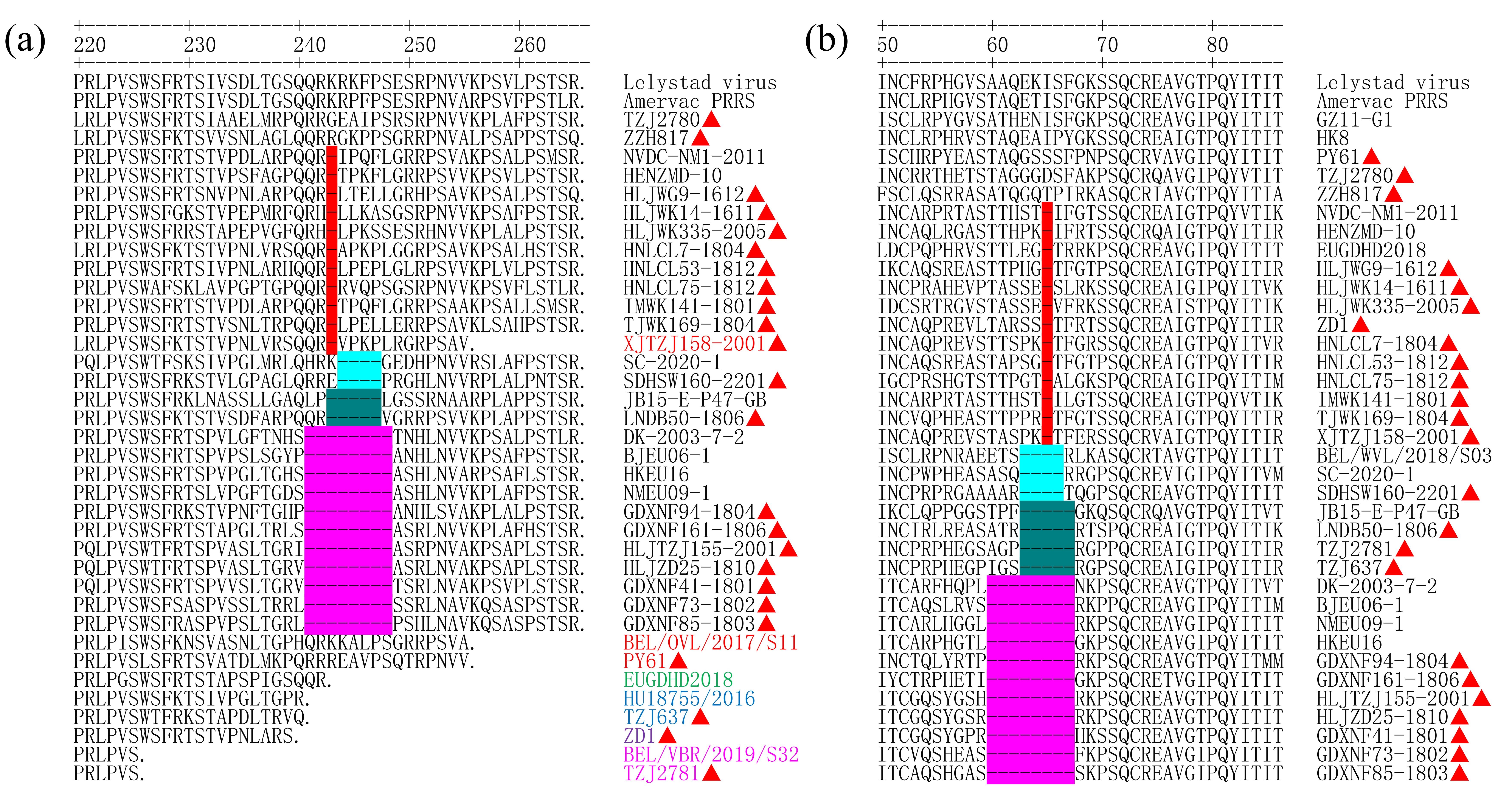


Fig. S1 Amino acid alignment of GP3 (a) and GP4 (b). The four different amino acid deletion patterns are shadowed in red, turquoise, cyan, and pink. The red triangle (▲) represents the twenty-four strains identified in this study. The five premature termination patterns of PRRSV-1 are marked in red, green, blue, purple, and pink.
