# Supplementary material for: Dissecting Genetic Diversity and Evolutionary Trends of Chinese PRRSV-1 Based on Whole-Genome Analysis
Source: Transbound Emerg Dis. 2024 Jun 11;2024:9705539. doi: 10.1155/2024/9705539 (PMC12017348; doi:10.1155/2024/9705539)
Supplement: Supplementary 4 — Figure 2: recombination analysis of strains GDXNF41-1801, HJLZD25-1810, GDXNF73-1802, GDXNF85-1803, and HKEU16. [file 9705539.f4.docx]

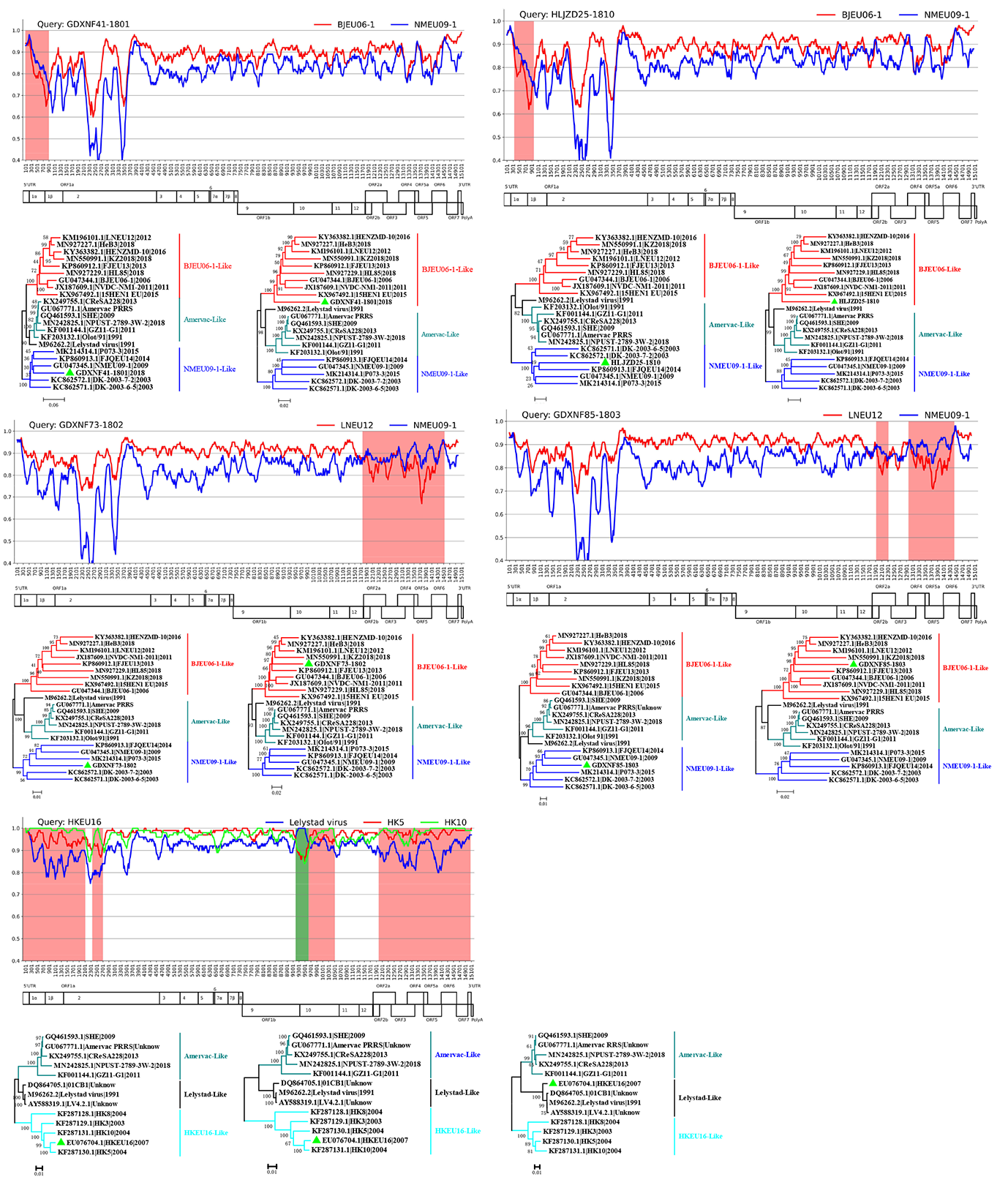


Fig. S2 Recombination analysis of strains GDXNF41-1801, HJLZD25-1810, GDXNF73-1802, GDXNF85-1803 and HKEU16. Similarity comparisons were performed using GDXNF41-1801, HJLZD25-1810, GDXNF73-1802, GDXNF85-1803 and HKEU16 as the query sequences, with LNEU12 (red), BJEU06-1 (red) or HK5 (red) as the major parent and NMEU09-1 (blue), HK10 (bright green) and/or Lelystad virus (blue) as the minor parent. The white background color represents the major parental region, while the light red and green background colors represent the minor parental region. The complete genome structure of PRRS-FR-2005-29-24-1 is shown below the similarity plots, following the phylogenetic tree of major and minor parental strains with five query strains marked by bright green triangles.
