# Supplementary material for: Dissecting Genetic Diversity and Evolutionary Trends of Chinese PRRSV-1 Based on Whole-Genome Analysis
Source: Transbound Emerg Dis. 2024 Jun 11;2024:9705539. doi: 10.1155/2024/9705539 (PMC12017348; doi:10.1155/2024/9705539)
Supplement: Supplementary 6 — Figure 3: estimation of the median time to the most recent common ancestor (tMRCA) for PRRSV-1 strains from China based on the ORF5 gene. [file 9705539.f6.docx]

Fig. S3 Estimation of the median time to the most recent common ancestor (tMRCA) for PRRSV-1 strains from China based on the ORF5 gene. The numbers near the internal node indicate the estimated median time of divergence. The numbers in square brackets represent the 95% HPD range of node divergence time. (a) Time-scaled phylogeny of the PRRSV-1 strains from China ORF5 gene. This maximum clade credibility (MCC) tree was generated via the MCMC method using BAEST (version 1.10.4) under uncorrelated lognormal relaxed molecular clocks and the Bayesian Skyline demographic model. (b) Time-scaled phylogeny of the BJEU06-1-Like PRRSV ORF5 gene. This maximum clade credibility (MCC) tree was generated via the MCMC method using BAEST (version 1.10.4) under strict molecular clocks and the Bayesian Skyline demographic model.
