# Supplementary material for: Dissecting Genetic Diversity and Evolutionary Trends of Chinese PRRSV-1 Based on Whole-Genome Analysis
Source: Transbound Emerg Dis. 2024 Jun 11;2024:9705539. doi: 10.1155/2024/9705539 (PMC12017348; doi:10.1155/2024/9705539)
Supplement: Supplementary 7 — Table 4: marginal likelihoods estimated by molecular clock models and coalescent models for BJEU06-1-Like PRRSV. [file 9705539.f7.docx]

**Table S4 Marginal likelihoods estimated by molecular clock models and coalescent models for BJEU06-1-Like PRRSV.**

| **Clock models** | **Coalescent models** | **Mean value of Log marginal likelihood** | **Rank** |
| --- | --- | --- | --- |
| Uncorrelated exponential relaxed clock | Bayesian Skyline | -4200.684906 | 11 |
| Uncorrelated exponential relaxed clock | Constant Size | -4206.383685 | 15 |
| Uncorrelated exponential relaxed clock | Expansion Growth | -4206.081116 | 14 |
| Uncorrelated exponential relaxed clock | Exponential Growth | -4200.909234 | 12 |
| Uncorrelated exponential relaxed clock | Logistic Growth | -4199.380743 | 9 |
| Uncorrelated lognormal relaxed clock | Bayesian Skyline | -4190.519021 | 4 |
| Uncorrelated lognormal relaxed clock | Constant Size | -4202.764199 | 13 |
| Uncorrelated lognormal relaxed clock | Expansion Growth | -4198.411742 | 8 |
| Uncorrelated lognormal relaxed clock | Exponential Growth | -4195.9841 | 6 |
| Uncorrelated lognormal relaxed clock | Logistic Growth | -4194.38957 | 5 |
| **Strict clock** | **Bayesian Skyline** | **-4184.95564** | **1** |
| Strict clock | Constant Size | -4200.467205 | 10 |
| Strict clock | Expansion Growth | -4197.698778 | 7 |
| Strict clock | Exponential Growth | -4190.359858 | 3 |
| Strict clock | Logistic Growth | -4189.979681 | 2 |

To obtain the mean values of the log marginal likelihood (using path sampling), every combination of molecular clock models and coalescent tree priors was independently run three times via path sampling and stepping-stone sampling procedures. The best-fitting combinations of molecular clock models and coalescent tree priors are indicated in bold font.
